# Challenging prior assumptions: coexistence of polycystic ovarian morphology and endometriosis on transvaginal ultrasound

**DOI:** 10.1002/uog.70289

**Published:** 2026-07-31

**Authors:** P. Romeo, S. M. Freger, N. Kalani, A. Shishkina, S. Aharoni, R. Shetty, M. Leonardi

**Affiliations:** ^1^ Obstetrics and Gynecology McMaster University Faculty of Health Sciences Hamilton ON Canada; ^2^ Gynecology and Obstetrics Unit, Department of Human Pathology of Adult and Childhood “G. Barresi” University of Messina Messina Italy

**Keywords:** endometriosis, PCOM, PCOS, polyendocrine metabolic ovarian syndrome, polycystic ovarian morphology, polycystic ovary syndrome, PMOS, transvaginal ultrasound

## Abstract

**Objective:**

To investigate, using transvaginal ultrasound imaging, the overlap between polycystic ovarian morphology (PCOM) and endometriosis, and to explore demographic and clinical factors associated with their coexistence across endometriosis phenotypes.

**Methods:**

This was a retrospective cohort study of consecutive patients undergoing advanced transvaginal ultrasound examination for suspected or previously diagnosed endometriosis at a tertiary gynecological ultrasound clinic between February 2023 and June 2023. Ultrasound was performed following the International Deep Endometriosis Analysis consensus and the International Ovarian Tumor Analysis framework guided lesion characterization, with PCOM defined as per the 2018 and the modified 2023 International Polycystic Ovary Syndrome Guideline (≥ 20 follicles measuring 2–9 mm in diameter and/or ovarian volume > 10 mL in the absence of a dominant follicle, cyst or corpus luteum). Endometriosis phenotypes were classified sonographically as superficial (SE), ovarian (OE) or deep (DE) endometriosis. Demographic and clinical variables were compared between participants with endometriosis alone and those with concurrent endometriosis and PCOM. Logistic regression analysis was performed within the endometriosis‐positive subgroup using PCOM as the dependent variable in a univariable model including age and a multivariable model including age and body mass index (BMI). An age‐restricted (25–35 years) sensitivity analysis compared the frequencies of any endometriosis and of individual endometriosis phenotypes between participants with and those without PCOM. A secondary analysis within this subgroup compared clinical and demographic characteristics between endometriosis participants with and those without concurrent PCOM.

**Results:**

Among 165 included patients, 62.4% (*n* = 103) were diagnosed with endometriosis, 37.0% (*n* = 61) demonstrated PCOM and 35.0% (36/103) of those with endometriosis had concurrent PCOM. In the whole cohort, the frequency of any endometriosis did not differ significantly between participants with and those without PCOM. DE was less frequent among those with PCOM, whereas differences in the frequency of OE and SE between participants with and those without PCOM were not statistically significant. Compared to individuals with endometriosis alone, those with concurrent PCOM were significantly younger and had lower body weight. The proportions of nulligravidae and of nulliparae were also higher. In age‐restricted analyses limited to participants aged 25–35 years, no significant differences were observed between those with and those without PCOM for the presence of any endometriosis or for any individual endometriosis phenotype. However, among endometriosis‐positive participants, increasing age was associated with lower odds of concurrent PCOM in both the univariable model including age (odds ratio (OR), 0.83 (95% CI, 0.77–0.90) per 1‐year increase; *P* < 0.001) and the multivariable model including age and BMI (OR, 0.84 (95% CI, 0.77–0.92) per 1‐year increase; *P* < 0.001), whereas BMI was not independently associated with PCOM (OR, 0.99 (95% CI, 0.91–1.09) per 1 kg/m^2^ increase; *P* = 0.874). In the sensitivity analysis restricted to participants aged 25–35 years, the differences observed previously in age, weight, gravidity and parity were no longer statistically significant.

**Conclusions:**

Endometriosis and PCOM can coexist on ultrasound in a tertiary referral cohort. However, the observed between‐group differences were strongly influenced by age, and our findings should be interpreted as exploratory and hypothesis‐generating rather than confirmatory. Prospective studies incorporating endocrine characterization, standardized cycle‐phase assessment and side‐specific ovarian assessment are needed to clarify the clinical and biological significance of this coexistence. © 2026 The Author(s). *Ultrasound in Obstetrics & Gynecology* published by John Wiley & Sons Ltd on behalf of International Society of Ultrasound in Obstetrics and Gynecology.

## INTRODUCTION

Endometriosis and polycystic ovary syndrome (PCOS), now known as polyendocrine metabolic ovarian syndrome (PMOS), are among the most prevalent gynecological conditions affecting individuals of reproductive age, and are associated with significant reproductive, hormonal and quality‐of‐life impact^1^, ^2^. Recent conceptual frameworks have proposed that these conditions may represent biologically divergent reproductive phenotypes, sometimes described as ‘diametric disorders’^3^, ^4^, ^5^. This model suggests that differences in prenatal androgen exposure and reproductive‐axis development may contribute to contrasting hormonal profiles in adulthood. Specifically, higher fetal androgen exposure is proposed to contribute to the hyperandrogenism characteristic of PMOS, whereas lower fetal androgen exposure is proposed to contribute to the relative hypoandrogenism associated with endometriosis^3^, ^4^, ^5^. Consistent with this developmental model, a longer anogenital distance was observed in newborn daughters of women with PMOS, whereas a shorter anogenital distance was observed in adults with endometriosis, relative to controls^6^. Furthermore, genetic studies have shown that higher testosterone‐related polygenic scores are associated with increased PMOS risk and reduced endometriosis risk^7^.

Endometriosis is a chronic, estrogen‐dependent disease characterized by ectopic endometrial‐like glands and stroma that lead to pain, infertility and inflammatory sequelae, affecting approximately 1 in 10 people assigned female sex at birth and gender diverse individuals globally^1^. Polycystic ovarian morphology (PCOM) is a sonographic descriptor defined as increased antral follicle number and/or ovarian volume in the absence of a dominant follicle, cyst or corpus luteum^2^. Although commonly associated with PMOS, PCOM can also occur in ovulatory and metabolically normal individuals, and does not necessarily reflect endocrine dysfunction^8^, ^9^, ^10^.

Accordingly, the present study aimed to examine the overlap between PCOM and endometriosis in a consecutive cohort of individuals referred for advanced transvaginal ultrasound examination. Using standardized sonographic frameworks, we sought to characterize how PCOM presents across distinct endometriosis phenotypes and to identify the demographic and clinical factors associated with the concurrent expression of PCOM. Our current hypothesis was informed by our prior work demonstrating an association between PCOM and dysmenorrhea^11^. Given that pelvic pain has also been reported in PMOS^12^, ^13^, we hypothesized that PCOM would be a common feature among people with suspected or previously diagnosed endometriosis.

## METHODS

### Study design and population

This retrospective cohort study was conducted at McMaster University, with participants recruited from a tertiary gynecological ultrasound clinic (Specialized Ultrasound Gynecology and Obstetrics (SUGO)) in Hamilton, ON, Canada. The study included consecutive patients referred to the SUGO clinic for advanced transvaginal ultrasound examination between February 2023 and June 2023 owing to suspected or previously diagnosed endometriosis. In this setting, ‘suspected endometriosis’ refers to referrals made by healthcare providers (i.e. gynecologist, primary care physician or nurse practitioner) based on clinical evaluation, most commonly for chronic pelvic pain, dysmenorrhea, dyspareunia, infertility or previous abnormal pelvic imaging suggestive of endometriosis. Participants were eligible for inclusion if they had completed the standardized intake questionnaire before the ultrasound scan and if both ovaries had been visualized. Exclusion criteria included lack of transvaginal ultrasound assessment, incomplete clinical data or onset of menarche within the preceding 8 years, in alignment with international PCOS guideline recommendations against the sonographic characterization of an ovary as ‘polycystic’ in this timeframe^14^. As this was an imaging‐based referral cohort rather than a population‐based sample, inclusion was determined by the clinical referral indication rather than standardized symptom criteria. The sample encompassed all consecutive eligible patients within the specified period; as an exploratory prevalence study, no formal sample‐size calculation was performed. The study protocol was reviewed and approved by the Hamilton Integrated Research Ethics Board (HiREB protocol number: 17389) as of February 2024, and the study was performed and reported in accordance with the Strengthening the Reporting of Observational Studies in Epidemiology (STROBE) guidelines^15^.

### Ultrasound acquisition and interpretation

All examinations were performed using high‐resolution transvaginal ultrasound systems under the supervision of, and reported by, an expert gynecological surgeon and sonologist (M.L.), accredited at European Federation of Societies for Ultrasound in Medicine and Biology Level 3. Ultrasound scans were performed by fellows in the McMaster University Minimally Invasive Gynecologic Surgery and Ultrasound fellowship program. Scanning protocols followed the International Deep Endometriosis Analysis (IDEA) consensus for systematic evaluation of the uterus, ovaries and posterior and anterior pelvic compartments^16^, incorporating peritoneal mapping for superficial endometriosis (SE)^17^, ^18^. Although the 2025 IDEA addendum on SE^17^ had not yet been published at the time of ultrasound acquisition, the principles from this addendum were already being applied in our clinic, and the characterization of SE features on ultrasound was also informed by previously published studies^19^.

Ovarian morphology was assessed in sagittal and transverse planes, with three‐dimensional measurements used to calculate ovarian volume. Follicle number per ovary was determined in real time by the ultasound operator and subsequently verified by the interpreting physician (M.L.) using stored cine loops and still frames. In cases involving cystic or solid adnexal lesions, the International Ovarian Tumor Analysis (IOTA) framework guided lesion characterization^20^. Although image acquisition and follicle counting were performed according to the international ultrasound criteria in use at the time of scanning (2018 PCOS Guideline)^21^, the 2023 International Evidence‐Based Guideline for the Assessment and Management of Polycystic Ovary Syndrome retained identical follicle count and ovarian volume thresholds^14^. PCOM was therefore defined as the presence of ≥ 20 follicles measuring 2–9 mm in diameter and/or an ovarian volume > 10 mL in the absence of a dominant follicle, cyst or corpus luteum.

Endometriosis was classified sonographically as present or absent. When present, it was further categorized as SE, ovarian endometriosis (OE) or deep endometriosis (DE), with subtype findings recorded independently as present or absent; therefore, these categories were not mutually exclusive, and a patient could be classified as having more than one subtype. In cases in which large ovarian cysts or endometriomas limited reliable follicle visualization, follicle counts were interpreted cautiously, and ovarian morphology was assessed using the contralateral ovary when feasible. The laterality of OE was not systematically recorded in the retrospective dataset; therefore, unilateral and bilateral OE could not be distinguished retrospectively for separate analysis or exclusion. As the study was conducted in a diagnostic imaging clinic providing ultrasound services for referring healthcare providers, including physicians and nurse practitioners, hormonal assays and metabolic indices were not performed; therefore, the full diagnostic criteria for PMOS could not be applied.

### Clinical data collection and variable definitions

Demographic and clinical data, including patient age, menstrual characteristics, reproductive history, prior abdominal or pelvic surgery and gynecological comorbidities, were self‐reported via standardized intake forms completed immediately before the ultrasound examination. The severity of dysmenorrhea and dyspareunia was recorded using an 11‐point numeric rating scale (0 to 10). Menstrual regularity was recorded as a binary variable (regular or irregular) with quantitative documentation of average cycle frequency.

### Statistical analysis

Continuous variables are presented as mean ± SD or median (interquartile range), as appropriate, and categorical variables are presented as *n* (%). The prevalence of PCOM was calculated for the entire cohort and stratified by the presence and subtype of endometriosis. Descriptive characteristics of the cohort were summarized, with exploratory between‐group comparisons conducted for contextual interpretation. Primary inferential analyses and secondary analysis using demographic and clinical variables compared individuals with endometriosis alone *vs* those with concurrent PCOM. Additional analyses compared the frequency of specific endometriosis phenotypes and clinical and demographic factors between participants with and those without PCOM in both the whole cohort and in a sensitivity analysis within an age‐restricted subgroup (25–35 years). The independent two‐sample *t*‐test was used for continuous variables and the chi‐square test was used for categorical variables; Fisher's exact test was used when expected cell counts were < 5. To address potential confounding by age and body mass index (BMI), logistic regression analysis was performed within the endometriosis‐positive subgroup, with PCOM as the dependent variable in a univariable model including age and a multivariable model including age and BMI. Analysis was conducted using IBM SPSS Statistics version 28 (IBM Corp., Armonk, NY, USA); statistical significance was defined as *P* < 0.05. As the primary objective was to describe the coexistence of PCOM in a consecutive ultrasound‐based endometriosis cohort, this study was considered exploratory and hypothesis‐generating.

## RESULTS

### Cohort characteristics

**Figure 1:**
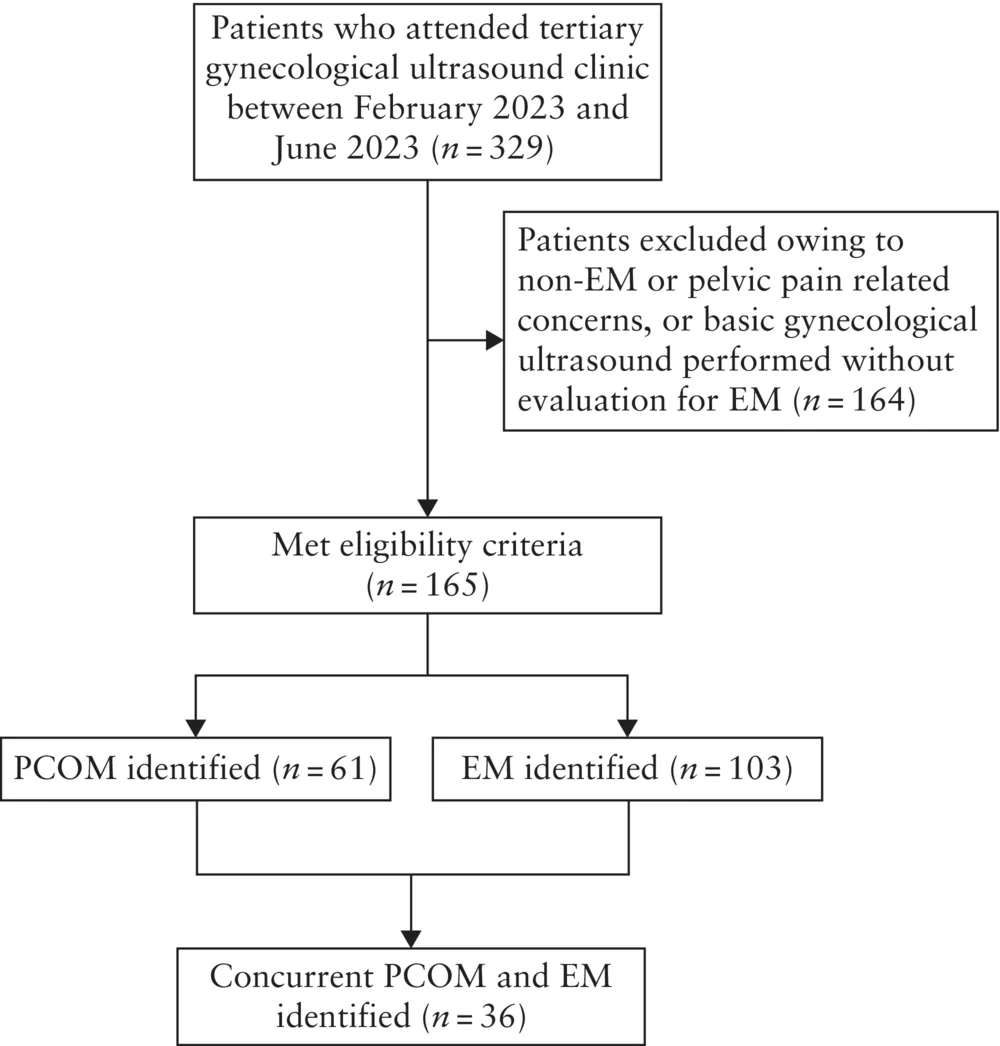
Flowchart showing inclusion of study participants and distribution of patients diagnosed with polycystic ovarian morphology (PCOM) and/or endometriosis (EM). In 37 cases, neither PCOM nor EM was diagnosed.

During the study period, 165 consecutive patients underwent advanced transvaginal ultrasound imaging for suspected or previously diagnosed endometriosis, including referrals prompted by pelvic pain, dysmenorrhea, dyspareunia, infertility or previous abnormal pelvic imaging, and completed standardized clinical intake forms. These patients formed the analytic cohort (Figure 1). PCOM was identified in 37.0% (*n* = 61) of participants, and 62.4% (*n* = 103) had an ultrasound‐based diagnosis of endometriosis. The co‐occurrence of PCOM was noted in 35.0% (36/103) of participants with endometriosis. Among those with PCOM, endometriosis was also noted in 59.0% (36/61). Overall, PCOM in the absence of endometriosis was observed in 15.2% (25/165) of patients, whereas endometriosis without PCOM was identified in 40.6% (67/165). Notably, 22.4% (37/165) of participants showed no sonographic evidence of either PCOM or endometriosis.

In the whole cohort, the overall frequency of any endometriosis did not differ significantly between participants with and those without PCOM (59.0% (36/61) *vs* 64.4% (67/104); *P* = 0.509). DE was less frequent among participants with PCOM (14.8% (9/61) *vs* 38.5% (40/104); *P* = 0.001), whereas differences in OE (6.6% (4/61) *vs* 16.3% (17/104); *P* = 0.090) and SE (45.9% (28/61) *vs* 32.7% (34/104); *P* = 0.099) were not statistically significant (Table 1). In the age‐restricted sensitivity analysis of participants aged 25–35 years, the presence of any endometriosis and of DE, OE and SE individually did not differ significantly between participants with and those without PCOM (Table 1). Descriptive characteristics of the cohort are summarized in Table 2, with exploratory between‐group comparisons provided for contextual interpretation.

**Table 1:** Frequency of endometriosis overall and of individual endometriosis phenotypes in participants with and without polycystic ovarian morphology (PCOM) in whole cohort and in age‐restricted subgroup (25–35 years)

Endometriosis phenotype	Total	With PCOM	Without PCOM	*P*
Whole population	165	61	104	
Any endometriosis	103/165 (62.4)	36/61 (59.0)	67/104 (64.4)	0.509
DE	49/165 (29.7)	9/61 (14.8)	40/104 (38.5)	0.001
OE	21/165 (12.7)	4/61 (6.6)	17/104 (16.3)	0.090
SE	62/165 (37.6)	28/61 (45.9)	34/104 (32.7)	0.099
Age‐restricted subgroup (25–35 years)	72	39	33	
Any endometriosis	45/72 (62.5)	21/39 (53.8)	24/33 (72.7)	0.143
DE	15/72 (20.8)	5/39 (12.8)	10/33 (30.3)	0.086
OE	7/72 (9.7)	3/39 (7.7)	4/33 (12.1)	0.695
SE	32/72 (44.4)	17/39 (43.6)	15/33 (45.5)	1.000

Data are given as *n*, *n* (%) or *n*/*N* (%). *P*‐values represent two‐sided Fisher's exact tests comparing participants with PCOM *vs* those without PCOM. Specific endometriosis phenotypes were recorded as non‐mutually exclusive. Therefore, participants with more than one phenotype, such as concurrent superficial (SE), ovarian (OE) and/or deep (DE) endometriosis, were counted once in the ‘any endometriosis’ category and separately within each applicable phenotype‐specific category.

**Table 2:** Demographic and clinical characteristics of study cohort according to endometriosis and polycystic ovarian morphology (PCOM) status

Characteristic	None (*n* = 37)	PCOM only (*n* = 25)	Endometriosis only (*n* = 67)	Endometriosis and PCOM (*n* = 36)	*P*
Demographic					
Age (years)	37.2 ± 8.9	30.9 ± 4.9	38.2 ± 8.8	28.1 ± 5.7	< 0.0001
Age at menarche (years)	12.3 ± 1.6	12.3 ± 1.5	12.4 ± 1.8	12.5 ± 1.5	0.951
Height (cm)	165.3 ± 7.1	164.2 ± 7.4	165.1 ± 6.6	165.3 ± 7.6	0.928
Weight (kg)	70.5 ± 14.9	71.9 ± 16.2	74.2 ± 18.6	64.9 ± 14.8	0.064
BMI (kg/m^2^)	25.7 ± 5.3	27.1 ± 7.1	27.1 ± 7.6	24.2 ± 4.9	0.154
Reproductive history					
Gravidity					0.047
0	15 (40.5)	15 (60.0)	31 (46.3)	27 (75.0)	
1	5 (13.5)	5 (20.0)	12 (17.9)	6 (16.7)	
2	7 (18.9)	1 (4.0)	11 (16.4)	2 (5.6)	
≥ 3	10 (27.0)	4 (16.0)	13 (19.4)	1 (2.8)	
Parity					0.010
0	18 (48.6)	17 (68.0)	41 (61.2)	33 (91.7)	
1	8 (21.6)	4 (16.0)	7 (10.4)	3 (8.3)	
2	7 (18.9)	4 (16.0)	12 (17.9)	0 (0)	
≥ 3	4 (10.8)	0 (0)	7 (10.4)	0 (0)	
History of infertility	6 (16.2)	5 (20.0)	16 (23.9)	7 (19.4)	0.823
Menstrual					
Length between cycle (days)	28 (25–30)	29 (27–36)	29 (25–30)	29 (26–32)	0.546
Duration of bleeding (days)	6 (5–7)	6 (5–9)	6 (5–7)	7 (5–9)	0.619
Menstrual regularity					0.022
Regular	24 (64.9)	8 (32.0)	44 (65.7)	19 (52.8)	
Irregular	13 (35.1)	17 (68.0)	23 (34.3)	17 (47.2)	
Flow intensity					0.093
Light	17 (45.9)	4 (16.0)	22 (32.8)	8 (22.2)	
Moderate	6 (16.2)	6 (24.0)	20 (29.9)	13 (36.1)	
Severe	14 (37.8)	15 (60.0)	25 (37.3)	15 (41.7)	
Pain and symptomatology					
Dysmenorrhea	8 (7–10)	10 (8–10)	8 (7–10)	9 (6.5–10)	0.351
Dyspareunia	5 (3–8)	5 (4–6)	5 (3–7)	6 (4–8)	0.178
Surgical and clinical history					
Previous pelvic surgery (non‐endometriosis)	12 (32.4)	3 (12.0)	19 (28.4)	4 (11.1)	0.057
Previous endometriosis surgery	6 (16.2)	2 (8.0)	17 (25.4)	3 (8.3)	0.082

Data are given as mean ± SD, *n* (%) or median (interquartile range). *P*‐values represent exploratory comparisons across the four study groups and were calculated using one‐way ANOVA for parametric continuous variables, Kruskal–Wallis tests for non‐parametric continuous variables and chi‐square or Fisher's exact tests for categorical variables, as appropriate. BMI, body mass index.

### Inferential comparisons among individuals with endometriosis

Inferential analyses comparing individuals with endometriosis alone and those with concurrent PCOM are presented in Table S1. Individuals with concurrent PCOM were significantly younger than those with endometriosis alone (*P* < 0.00001). Body weight was significantly lower among those with concurrent PCOM (*P* = 0.028), whereas BMI showed a similar trend but the association did not reach statistical significance (*P* = 0.061). No significant differences were observed for age at menarche or height. Reproductive history differed between the groups. The gravidity distribution was significantly different between individuals with and those without concurrent PCOM (*P* = 0.015), with a higher proportion of nulligravid individuals among those with concurrent PCOM. Parity showed a similar pattern, with nulliparity being significantly more common in the group with concurrent PCOM (*P* = 0.004). Menstrual characteristics did not differ significantly between the groups, including cycle length (*P* = 0.370), duration of bleeding (*P* = 0.149), menstrual regularity (*P* = 0.285), flow intensity (*P* = 0.520) and a history of infertility (*P* = 0.789). With respect to surgical history, prior non‐endometriosis pelvic surgery was less frequent among individuals with concurrent PCOM, although this difference did not reach statistical significance (*P* = 0.079). Prior endometriosis surgery was significantly less common in the group with concurrent PCOM compared to individuals with endometriosis alone (*P* = 0.040; Fisher's exact test).

To address potential confounding by age and BMI, logistic regression analysis was performed within the endometriosis‐positive subgroup using PCOM as the dependent variable. In the univariable model including age, increasing age was associated with lower odds of concurrent PCOM (odds ratio (OR), 0.83 (95% CI, 0.77–0.90) per 1‐year increase; *P* < 0.001). In the multivariable model including age and BMI, age remained independently associated with PCOM (OR, 0.84 (95% CI, 0.77–0.92) per 1‐year increase; *P* < 0.001), whereas BMI was not independently associated with PCOM (OR, 0.99 (95% CI, 0.91–1.09) per 1 kg/m^2^ increase; *P* = 0.874). In a sensitivity analysis restricted to participants aged 25–35 years, the differences in age, weight, gravidity and parity observed previously were attenuated and were no longer statistically significant (Table S2). No significant between‐group differences were observed in infertility history, menstrual characteristics or surgical history in this restricted analysis.

## DISCUSSION

In this cohort of patients referred for tertiary ultrasound assessment, PCOM and sonographically detected endometriosis frequently coexisted. Approximately one‐third of participants with endometriosis had concurrent PCOM. However, the additional analyses indicated that age, rather than a distinct clinical profile, was the principal factor associated with concurrent PCOM. Increasing age remained associated with lower odds of concurrent PCOM after adjustment for BMI, while BMI was not independently associated with PCOM. Moreover, several crude between‐group differences, including differences in age, weight, gravidity and parity, were attenuated in the age‐restricted sensitivity analysis. Together, these findings suggest that the coexistence of PCOM and endometriosis can be identified on ultrasound; however, cross‐sectional differences between groups should be interpreted cautiously and considered primarily as exploratory.

These findings add nuance to prior conceptual models that have framed endometriosis and PMOS as biologically divergent reproductive phenotypes^3^, ^4^, ^5^, ^19^, ^22^. Although endometriosis has often been discussed in relation to relative hypoandrogenism and PMOS in relation to hyperandrogenism^23^, ^24^, our results do not support a simple mutually exclusive relationship at the level of ovarian morphology. At the same time, the present study assessed PCOM rather than PMOS. This distinction is critical, as PCOM may occur in ovulatory, normoandrogenic and metabolically normal individuals and therefore should not be interpreted as equivalent to syndrome‐level endocrine dysfunction^2^. Our findings therefore support the coexistence of sonographic PCOM and endometriosis within a referral population, but do not directly challenge or confirm broader pathophysiological models regarding PMOS and endometriosis.

Our age‐related findings are particularly important for interpretation. In the whole‐cohort analysis, DE occurred less frequently among participants with PCOM, whereas SE was numerically more frequent, albeit this difference did not reach statistical significance. However, within the age‐restricted sensitivity analysis, the distribution of endometriosis phenotypes was broadly similar between participants with and those without PCOM, and no phenotype‐specific differences reached statistical significance. This suggests that at least some apparent phenotype‐level differences may reflect age structure within the cohort rather than a true association between PCOM and specific endometriosis subtypes. Additionally, the lower frequency of DE among participants with PCOM (9/61 (14.8%)) compared with those without PCOM (40/104 (38.5%)), together with a similar directional pattern in the age‐restricted subgroup (12.8% (5/39) *vs* 30.3% (10/33)), may suggest that PCOM or its associated ovarian microenvironment is less susceptible to deep disease. This remains speculative, as the age‐restricted analysis was underpowered, and age‐related confounding cannot be excluded, warranting further study in larger cohorts with endocrine characterization. Given the known age‐related decline in antral follicle count^25^, ^26^, ^27^, this issue is also methodologically relevant because fixed ultrasound thresholds for PCOM may preferentially classify younger participants as having PCOM.

From a clinical imaging perspective, these findings support systematic ovarian assessment for PCOM during pelvic ultrasound examination, even when endometriosis is the primary indication for referral. Routine documentation of follicle number and ovarian volume, when technically feasible, may improve phenotypic characterization and reduce the risk of overlooking relevant ovarian morphology. However, such documentation should remain descriptive. In this setting, identification of PCOM should not be conflated with a diagnosis of PMOS, particularly when endocrine, ovulatory and metabolic data are unavailable.

### Strengths and limitations

A major strength of this work is the use of harmonized, internationally validated ultrasound frameworks, including the IDEA consensus for endometriosis mapping and IOTA descriptors and strict PMOS ultrasound guidelines for adnexal morphology, which enables clinical and research reproducibility. The cohort was consecutive and unselected within the referral population, minimizing operator and selection bias, and all scans were performed by highly trained gynecological ultrasound operators and interpreted by an expert gynecological sonologist, enhancing diagnostic consistency.

Several limitations warrant detailed consideration. First, the retrospective study design precludes causal inference and may have introduced residual or unmeasured confounding, particularly related to referral patterns and reproductive stage. Second, endocrine, metabolic and ovulatory parameters were not available, preventing differentiation between isolated PCOM and full PMOS and their respective phenotypes^28^; hormonal assays (androgens, luteinizing hormone/follicle‐stimulating hormone ratio, anti‐Müllerian hormone) would be required to delineate syndrome‐level overlap. Information on prior hormonal or endocrine treatments for endometriosis or PMOS was not systematically collected, and therefore potential effects of treatment exposure on ovarian morphology could not be evaluated. An additional limitation relates to the distinction between PCOM and PMOS. PCOM is relatively common in the general population and occurs more frequently than does PMOS, meaning ovarian morphology alone does not indicate endocrine or metabolic dysfunction. Accordingly, our findings describe the coexistence of PCOM, rather than PMOS, and endometriosis, and should not be interpreted as direct evidence regarding theoretical models addressing the relationship between PMOS and endometriosis.

The menstrual cycle phase at the time of imaging was not standardized; follicular‐phase scans may show higher antral counts than do luteal‐phase scans, which may affect PCOM classification^29^. Ovarian follicle counts may also have been influenced by disease‐related factors, as OE has been associated with reduced ovarian reserve^30^. Additionally, the presence of endometriomas may reduce the ability to visualize small follicles on ultrasound, potentially leading to underestimation of follicle counts in affected ovaries^31^, ^32^. In this retrospective dataset, the laterality of OE and side‐specific evaluability of follicle visualization were not systematically recorded; therefore, unilateral and bilateral ovarian disease could not be distinguished retrospectively, and the potential masking effect of bilateral OE could not be quantified directly. As a result, the reported prevalence of PCOM within the OE subgroup may have been underestimated and should be interpreted cautiously. Furthermore, referral for imaging was based on clinical suspicion rather than standardized symptom thresholds, and symptom severity does not necessarily correlate with the presence or extent of endometriosis lesions. Additionally, as the study was exploratory and no formal sample‐size calculation was performed, the sample size may have limited statistical power to detect smaller associations, particularly in the subgroup comparisons. Moreover, as endometriosis was assessed using ultrasound rather than surgical confirmation, imaging‐based diagnosis may have introduced misclassification, particularly for SE, or operator‐dependent variability, although standardized scanning protocols were applied. Finally, PCOM was defined using fixed 2018 guideline thresholds applied to a broad, age‐unbalanced reproductive cohort. Because antral follicle counts decline physiologically with age, applying an age‐independent threshold may bias PCOM classification toward younger participants and against older participants. Although we addressed this concern through regression analyses including age and an age‐restricted sensitivity analysis, residual age‐related diagnostic bias cannot be excluded.

### Conclusions

PCOM is a common sonographic finding among individuals undergoing transvaginal ultrasound evaluation for suspected or previously diagnosed endometriosis. In this cohort, PCOM and endometriosis frequently co‐occurred on ultrasound imaging; however, age was the dominant factor associated with concurrent PCOM, and several crude between‐group differences were attenuated after age restriction. These findings should therefore be interpreted as exploratory and hypothesis‐generating rather than confirmatory. Prospective studies incorporating endocrine characterization, standardized cycle‐phase assessment and side‐specific ovarian documentation are needed to determine whether concurrent PCOM has independent clinical or biological significance within endometriosis populations.

## Supporting information


**Table S1** Clinical characteristics associated with concurrent polycystic ovarian morphology (PCOM) in patients with endometriosis.

**Table S2** Age‐restricted (25–35 years) sensitivity analysis comparing participants with sonographically confirmed endometriosis with *vs* those without concurrent polycystic ovarian morphology (PCOM).

## Data Availability

The data that support the findings of this study are available from the corresponding author upon reasonable request.
