# Supplementary material for: Challenging prior assumptions: coexistence of polycystic ovarian morphology and endometriosis on transvaginal ultrasound
Source: Ultrasound Obstet Gynecol. 2026 Jul 31;68(3):406–12. doi: 10.1002/uog.70289 (PMC13532130; doi:10.1002/uog.70289)
Supplement: Supplementary file 1 [file UOG-68-406-s001.docx]

**Table S1.** Clinical characteristics associated with concurrent polycystic ovarian morphology (PCOM) in patients with endometriosis

| **Variable** | **Endometriosis only (n = 67)** | **Endometriosis + PCOM (n = 36)** | **p-value** |
| --- | --- | --- | --- |
| **Demographic and anthropometric** |  |  |  |
| Age (years), mean ± SD | 38.2 ± 8.8 | 27.2 ± 1.5 | **<0.00001** |
| Age at menarche (years), mean ± SD | 12.4 ± 1.8 | 12.5 ± 1.5 | 0.642 |
| Height (cm), mean ± SD | 165.1 ± 6.6 | 165.3 ± 7.6 | 0.866 |
| Weight (kg), mean ± SD | 74.2 ± 18.6 | 64.9 ± 14.8 | **0.028** |
| BMI (kg/m²), mean ± SD | 27.1 ± 7.6 | 24.2 ± 4.9 | 0.061 |
| **Reproductive history** |  |  |  |
| Gravidity, n (%) |  |  | **0.015** |
| 0 | 46.3 | 75.0 |  |
| 1 | 17.9 | 16.7 |  |
| 2 | 14.4 | 5.6 |  |
| ≥3 | 19.4 | 2.8 |  |
| Parity, n (%) |  |  | **0.004** |
| 0 | 61.2 | 91.7 |  |
| 1 | 10.4 | 8.3 |  |
| ≥2 | 28.4 | 0.0 |  |
| History of infertility, n (%) | 23.9 | 19.4 | 0.789 |
| **Menstrual features** |  |  |  |
| Cycle length (days), mean ± SD | 28.8 ± 8.2 | 29.0 ± 13.9 | 0.370 |
| Duration of bleeding (days), mean ± SD | 6.0 ± 3.7 | 7.0 ± 10.8 | 0.149 |
| Regular cycles, n (%) | 34.3 | 47.2 | 0.285 |
| Flow intensity, n (%) |  |  | 0.520 |
| Light | 32.8 | 22.2 |  |
| Moderate | 29.9 | 36.1 |  |
| Severe | 37.3 | 41.7 |  |
| **Surgical history** |  |  |  |
| Prior pelvic surgery (non-endometriosis), n (%) | 28.4 | 11.1 | 0.079 |
| Prior endometriosis surgery, n (%) | 25.4 | 8.3 | **0.040†** |

Data are presented as mean ± standard deviation for continuous variables and number (percentage) for categorical variables unless otherwise indicated. Comparisons were restricted to individuals with sonographically confirmed endometriosis. Two-sided independent samples t tests were used for continuous variables and χ² tests for categorical variables. Statistical significance was defined as p < 0.05. † Fisher’s exact test was used where expected cell counts were <5. P-values represent comparisons between endometriosis-only and endometriosis + PCOM groups.
