# Supplementary material for: Challenging prior assumptions: coexistence of polycystic ovarian morphology and endometriosis on transvaginal ultrasound
Source: Ultrasound Obstet Gynecol. 2026 Jul 31;68(3):406–12. doi: 10.1002/uog.70289 (PMC13532130; doi:10.1002/uog.70289)
Supplement: Supplementary file 2 [file UOG-68-406-s002.docx]

**Supplementary 2.** Age-restricted (25–35 years) sensitivity analysis comparing participants with sonographically confirmed endometriosis with and without concurrent polycystic ovarian morphology (PCOM).

| **Variable** | **Endometriosis only**  **(n = 24)** | **Endometriosis + PCOM (n = 21)** | **p-value** |
| --- | --- | --- | --- |
| **Demographic and anthropometric** |  |  |  |
| Age (years), mean ± SD | 30.4 ± 3.1 | 28.6 ± 3.2 | 0.071 |
| Age at menarche (years), mean ± SD | 12.1 ± 1.5 | 12.5 ± 1.4 | 0.336 |
| Height (cm), mean ± SD | 165.4 ± 9.3 | 167.8 ± 7.1 | 0.371 |
| Weight (kg), mean ± SD | 68.8 ± 22.5 | 66.2 ± 15.5 | 0.676 |
| BMI (kg/m²), mean ± SD | 24.7 ± 7.8 | 24.4 ± 4.7 | 0.862 |
| **Reproductive history** |  |  |  |
| Gravidity, (%) |  |  | 0.104 |
| *0* | 70.8 | 80.9 |  |
| *1* | 4.2 | 14.3 |  |
| *2* | 4.2 | 4.8 |  |
| *≥3* | 20.8 | 0.0 |  |
| Parity, (%) |  |  | 0.154 |
| *0* | 79.2 | 90.5 |  |
| *1* | 4.2 | 9.5 |  |
| *≥2* | 16.7 | 0.0 |  |
| History of infertility, (%) | 25.0 | 28.6 | 0.507 |
| **Menstrual features** |  |  |  |
| Cycle length (days), mean ± SD | 28.6 ± 7.0 | 32.3 ± 17.3 | 0.426 |
| Duration of bleeding (days), mean ± SD | 5.4 ± 2.0 | 8.1 ± 5.6 | 0.054 |
| Regular cycles, (%) | 33.3 | 28.6 | 0.757 |
| Flow intensity, (%) |  |  | 0.245 |
| *Light* | 16.7 | 28.6 |  |
| *Moderate* | 50.0 | 23.8 |  |
| *Severe* | 33.3 | 47.6 |  |
| **Surgical history** |  |  |  |
| Prior pelvic surgery (non-endometriosis), (%) | 16.7 | 14.3 | 0.678 |
| Prior endometriosis surgery, (%) | 12.5 | 14.3 | 0.307 |

Data are presented as mean ± standard deviation for continuous variables and n (%) for categorical variables unless otherwise indicated. Comparisons were restricted to participants aged 25–35 years with sonographically confirmed endometriosis and were performed as a sensitivity analysis to determine whether the differences observed in the primary endometriosis-only versus endometriosis + PCOM comparison persisted after age restriction. Two-sided independent-samples t tests were used for continuous variables, and χ² tests or Fisher’s exact tests, as appropriate, were used for categorical variables. Statistical significance was defined as p < 0.05. P-values represent comparisons between the endometriosis-only and endometriosis + PCOM groups.
